# Knockout of inositol-requiring enzyme 1α in pro-opiomelanocortin neurons decreases fat mass via increasing energy expenditure

**DOI:** 10.1098/rsob.160131

**Published:** 2016-08-24

**Authors:** Yuzhong Xiao, Tingting Xia, Junjie Yu, Yalan Deng, Hao Liu, Bin Liu, Shanghai Chen, Yong Liu, Feifan Guo

**Affiliations:** Key Laboratory of Nutrition and Metabolism, Institute for Nutritional Sciences, Shanghai Institute for Biological Sciences, Chinese Academy of Sciences, The Graduate School of the Chinese Academy of Sciences, 320 Yueyang Road, Shanghai, People's Republic of China

**Keywords:** hypothalamus, obesity, inositol-requiring enzyme 1α, leptin sensitivity, pro-opiomelanocortin neurons

## Abstract

Although numerous functions of inositol-requiring enzyme 1α (IRE1α) have been identified, a role of IRE1α in pro-opiomelanocortin (POMC) neurons in the arcuate nucleus of the hypothalamus is largely unknown. Here, we showed that mice lacking IRE1α specifically in POMC neurons (PIKO) are lean and resistant to high-fat diet-induced obesity and obesity-related insulin resistance, liver steatosis and leptin resistance. Furthermore, PIKO mice had higher energy expenditure, probably due to increased thermogenesis in brown adipose tissue. Additionally, α-melanocyte-stimulating hormone production was increased in the hypothalamus of PIKO mice. These results demonstrate that IRE1α in POMC neurons plays a critical role in the regulation of obesity and obesity-related metabolic disorders. Our results also suggest that IRE1α is not only an endoplasmic reticulum stress sensor, but also a new potential therapeutic target for obesity and obesity-related metabolic diseases.

## 1. Introduction

Energy balance relies on a tightly regulated homeostatic system matching food intake with energy expenditure [1]. The central nervous system (CNS), especially the hypothalamus, plays a key role in the regulation of energy homeostasis and insulin action [2]. The environmental and internal signals are integrated in the CNS to determine the different metabolic characteristics and phenotypes [3]. Because obesity and type 2 diabetes (T2D) represent an increasing health risk worldwide [4], exploring the nature of this dysfunction is high priority. Epidemiological studies have shown a relationship between dietary fat intake and obesity; therefore, high-fat diet (HFD)-induced obesity has been used commonly for the study of the mechanisms underlying dietary-induced obesity and insulin resistance [5].

In the hypothalamus, neurons expressing pro-opiomelanocortin (POMC), defined as part of the melanocortin system, play an important role in regulating food intake and energy expenditure [6], which is considered a promising target for the treatment of obesity [7].

Inositol-requiring enzyme 1α (IRE1α), as one member of three families of unique endoplasmic reticulum (ER) transmembrane proteins, is evolutionarily conserved in all eukaryotes from unicellular organisms to mammals [8] and widely expressed in brain and other tissues [9]. It contains a kinase domain and an RNase domain in the cytosolic region and is activated during ER adaptive response to stress [8,10–12]. The RNase activity induces splicing of X-box binding protein (XBP) 1 mRNA, which is translated into a functional transcription factor for ER stress control [13]. IRE1α is also activated through autophosphorylation to initiate a key signalling of the mammalian unfolded protein response (UPR) pathways [12,14]. It has been shown that IRE1α is involved in the regulation of many processes, including ER stress, autophagy and adipocyte differentiation [15–17]. However, a role of IRE1α in the hypothalamus has not been previously described.

In our current study, we demonstrated that mice lacking IRE1α specifically in POMC neurons are resistant to obesity and obesity-related metabolic disorders, the effects of which were mediated by increased energy expenditure. Interestingly, increased α-melanocyte-stimulating hormone (α-MSH) production was observed in the hypothalamus of POMC neuron-specific IRE1α knockout (PIKO) mice.

## 2. Material and methods

### 2.1. Animals and diets

C57BL/6 J wild-type (WT) mice were obtained from the Shanghai Laboratory Animals Co. Ltd (Shanghai, China). To generate PIKO mice, IRE1α floxed mice [18] were crossed with POMC-Cre mice [19]; all studies were performed in male PIKO mice and their littermate control mice. To visualize POMC-Cre positive neurons by immunofluorescence (IF) staining, POMC-Cre mice and PIKO mice were intercrossed with tdTomato reporter/Ai9 mice (The Jackson Laboratory) [20]. All animals were under C57BL/6 J background and housed in laboratory cages at 25 ± 2°C with a humidity of 35 ± 5% under a 12 h dark/light cycle, with free access to water and standard chow diet. For HFD experiments, male pups (*n* = 8–11) at ages of six weeks were fed a standard chow diet (NCD) or HFD with 60% kcal from fat (Research Diets, NJ, USA) for 16 weeks, then metabolic parameters, leptin sensitivity and glucose metabolism were measured in different cohorts of mice at the ages of 22–23 weeks old.

### 2.2. Metabolic parameter measurements

Indirect calorimetry was measured in a comprehensive laboratory animal monitoring system (CLAMS; Columbus Instruments, OH, USA), as previously described [21]. Rectal temperature of mice was measured at 14.00 (basal metabolic state) using a rectal probe attached to a digital thermometer (Physitemp, NJ, USA). Mice body composition was measured using NMR imaging system (Bruker, Rheinstetten, Germany).

### 2.3. Leptin sensitivity assay

Mice were injected intraperitoneally (i.p.) with either phosphate-buffered saline (PBS) or leptin (3 mg kg^−1^, R&D Systems, MN, USA) at 09.00 following fasting for 24 h as previously described [22]. Food intake and body weight were measured at 1 and 4 h post-injection of leptin or PBS.

### 2.4. Blood glucose, serum insulin, glucose tolerance tests, insulin tolerance tests and HOMA-IR

Levels of blood glucose and serum insulin were measured using a Glucometer Elite monitor and a Mercodia Ultrasensitive Rat Insulin ELISA kit (ALPCO Diagnostic, Salem, NH, USA), respectively. Glucose tolerance tests (GTTs) and insulin tolerance tests (ITTs) were performed by injection i.p. of 2 g kg^−1^ glucose after overnight fasting or 0.75 U kg^−1^ insulin after 4 h fasting, respectively. Homeostasis model assessment of insulin resistance (HOMA-IR) index was calculated according to the formula: fasting glucose levels × fasting serum insulin (μU ml^−1^)/22.5. Areas under curves (AUCs) were calculated as previously described [23].

### 2.5. Serum leptin, serum norepinephrine, serum corticosterone, serum growth hormone and hypothalamic alpha-melanocyte stimulating hormone measurement

Serum leptin, serum norepinephrine (NE), serum corticosterone and serum growth hormone levels were measured by a leptin ELISA kit (Merck Millipore, Darmstadt, Germany), an NE ELISA kit (Novus Biologicals, Littleton, USA), a corticosterone ELISA kit (Novus Biologicals) and a growth hormone ELISA kit (Merck Millipore), respectively, according to the manufacturer's instructions. Hypothalamic samples were prepared as previously described [24], and α-MSH was quantified by an ELISA kit (Phoenix Pharmaceuticals, CA, USA), according to the manufacturer's instructions.

### 2.6. Histological analysis of tissues

Paraformaldehyde-fixed, paraffin-embedded sections of white adipose tissue (WAT), brown adipose tissue (BAT) and liver were stained with haematoxylin–eosin (H&E) for histology. Frozen sections of liver were stained with Oil Red O.

### 2.7. Liver and serum triglyceride, cholesterol and free fatty acids measurements

Hepatic lipids were extracted by chloroform methanol (2 : 1) as previously described [25]. Hepatic and serum triglyceride (TG), cholesterol (TC) and free fatty acids (FFAs) were measured with a TG kit (SSUF-C, Shanghai, China), TC kit (SSUF-C) and FFA kit (Wako Pure Chemical Industries, Osaka, Japan), respectively, according to the manufacturer's instructions.

### 2.8. Immunofluorescence staining

IF staining with anti-IRE1α antibody (Novus Biologicals), anti-α-MSH antibody (Merck Millipore) and anti-ATF4 antibody (Santa Cruz Biotechnology, CA, USA) was performed as described previously [26]. p-STAT3 staining was performed as described previously [22]. All images were obtained using a Zeiss LSM 510 confocal microscope (Carl Zeiss Imaging, Oberkochen, Germany).

### 2.9. RNA isolation and relative quantitative RT–PCR

The RNA isolation and RT–PCR were performed as previously described [21]. The sequences of primers used in this study are available upon request.

### 2.10. Western blot analysis

Western blot was performed as previously described [21]. Primary antibodies, anti-IRE1α, anti-HSL, anti-p-HSL^Ser660^, anti-eIF2α, anti-p-eIF2α^Ser51^, anti-BiP and anti-p-PKA substrates (all from Cell Signaling Technology, MA, USA), anti-UCP1 and anti-ATF4 (Santa Cruz Biotechnology) and anti-β-actin (Sigma, MO, USA) were incubated at 4°C overnight, and specific proteins were visualized by ECL Plus (GE Healthcare, Buckinghamshire, UK).

### 2.11. Statistical analysis

All values are presented as means ± s.e.m. Differences between groups were analysed by either the Student *t*-test or one-way ANOVA followed by the Student–Newman–Keuls (SNK) test; differences in which *p* < 0.05 were considered statistically significant.

## 3. Results

### 3.1. PIKO mice are lean

**Figure 1.**
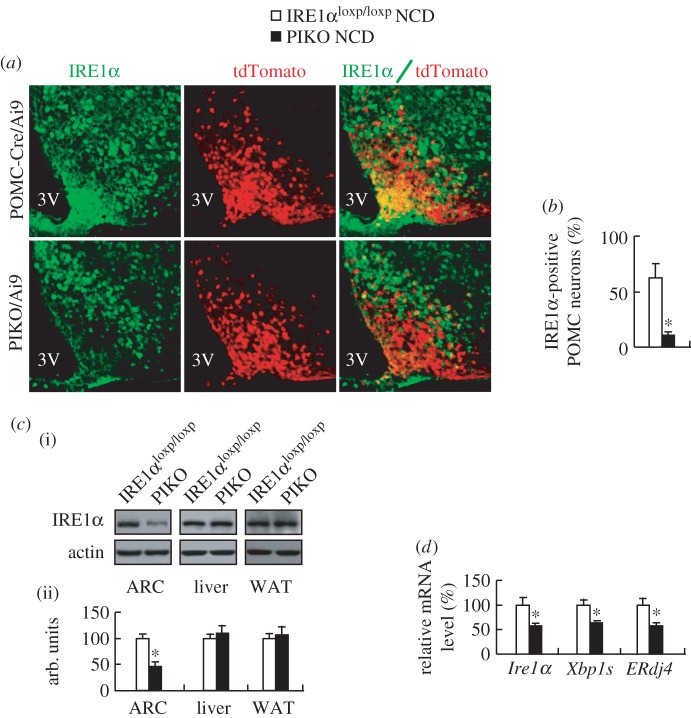
The generation of PIKO mice. (*a*) Representative IF images of the ARC from male POMC-Cre/Ai9 mice and PIKO/Ai9 mice, POMC neurons (red), IRE1α staining (green) and merge (yellow) (×20); 3V, third ventricle; (*b*) Statistical analysis of IRE1α-positive POMC neurons in ARC; (*c*) IRE1α protein in ARC, liver and WAT, (i, western blot; ii, quantitative measurement of IRE1α relative to actin); (*d*) *Ire1α, Xbp1s* and *ERdj4* mRNAs in ARC. All studies were conducted in eight-week-old male control and PIKO mice. Values are means ± s.e.m. (*n* = 4 per group in *a,b*, *n* = 8 per group in *c,d*), *p < 0.05.

To investigate the metabolic function of hypothalamic IRE1α, we generated PIKO mice. These mice were then intercrossed with Ai9 (tdTomato reporter) mice for examining the deletion efficiency of IRE1α in POMC neurons. IF staining showed that IRE1α was colocalized with POMC-expressing neurons in the arcuate nucleus (ARC) of control mice, but this colocalization was largely absent in PIKO mice (figure 1*a,b*). Furthermore, the expression of IRE1α and its downstream effectors including spliced *Xbp1* and *ERdj4* [18] was also decreased in the ARC of PIKO mice (figure 1*c,d*). Anatomical evaluation of the POMC neurons throughout the ARC of PIKO mice and control mice revealed no changes in neuronal population distribution and size (electronic supplementary material, figure S1), suggesting that IRE1α deficiency did not alter POMC neuron distribution and survival [27]. Furthermore, because POMC promoter also drives cre recombinase expression in the pituitary [27], we examined whether the function of the pituitary–adrenal axis and the basic phenotype of the pituitary were affected by IRE1α knockout. Serum contents of hormones secreted from the pituitary, including corticosterone and growth hormone [27–29], were comparable between PIKO mice and control mice (electronic supplementary material, figure S2*a,b*). Expression of pituitary genes, such as T box transcription factor (*Tpit*), *Pomc*, corticotrophin-releasing hormone receptor1 (*Crhr1*), growth hormone (*Gh*), pituitary-specific positive transcription factor 1 (*Pit1*) and thyroid-stimulating hormone β chain (*Tshb*) [27] were also not changed (electronic supplementary material, figure S2*c*). The size and neuronal distribution of the pituitary were also unaltered between PIKO mice and control mice (electronic supplementary material, figure S2*d,e*).

**Figure 2.**
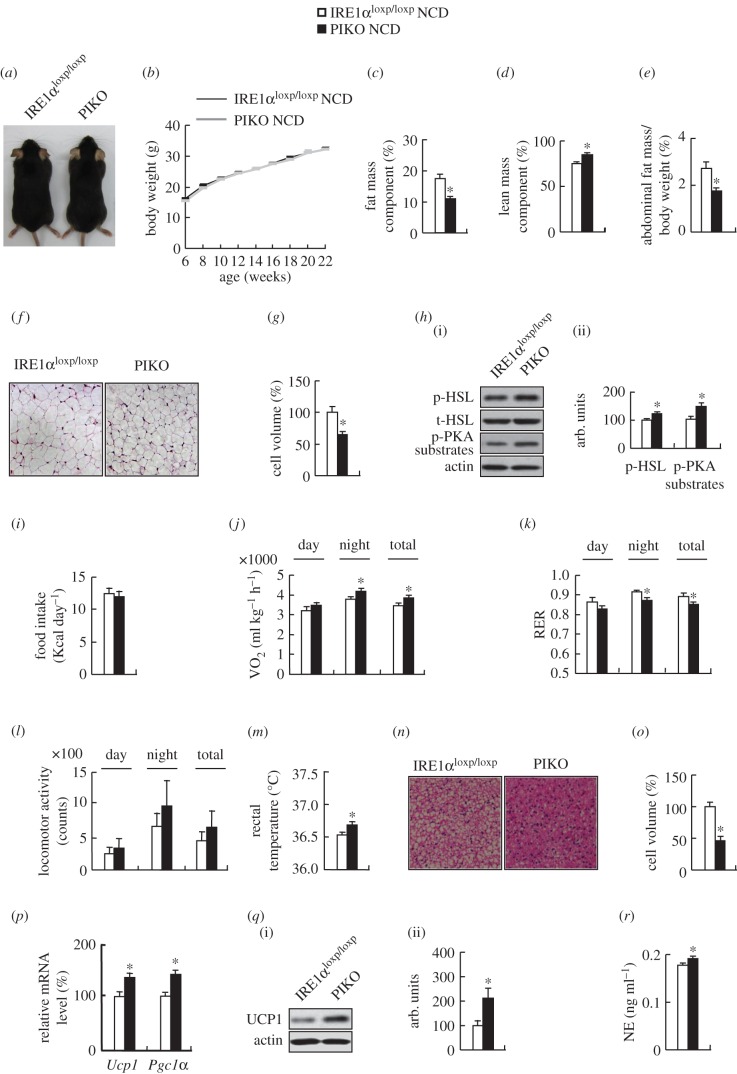
PIKO mice have reduced fat mass with increased energy expenditure. (*a*) The gross morphology; (*b*) body weight curve; (*c*) body fat mass component; (*d*) lean mass component; (*e*) abdominal fat mass; (*f*) representative images of H&E (haematoxylin and eosin) staining of abdominal WAT sections (×10); (*g*) the relative cell volume of WAT; (*h*) p-HSL, HSL and p-PKA substrate proteins in abdominal WAT (i, western blot; ii, quantitative measurements of p-HSL and p-PKA substrates relative to the total protein or actin); (*i*) food intake; (*j*) oxygen consumption; (*k*) respiratory exchange ratio (RER, 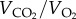); (*l*) locomotor activity; (*m*) average basal rectal temperature; (*n*) representative images of H&E staining of BAT sections (×10); (*o*) the relative cell volume of brown adipose tissue; (*p*) *Ucp1* and *Pgc1α* mRNAs in BAT; (*q*) UCP1 proteins in BAT (i, western blot; ii, quantitative measurement of UCP1 relative to actin); (*r*) serum norepinephrine (NE) levels. All studies were conducted in male control mice and PIKO mice maintained on a standard chow diet (NCD). Values are means ± s.e.m. (*n* = 8–10 per group), *p < 0.05.

Though gross morphology and body weight were not altered, body fat mass component and abdominal fat mass were significantly decreased and lean mass component was increased in PIKO mice compared with control mice maintained on a standard chow diet (figure 2*a–e*). Consistent with the reduced adiposity in PIKO mice, the adipocyte volume in WAT was significantly smaller, and phosphorylation of proteins related to lipolysis including hormone-sensitive lipase (HSL) and substrates of protein kinase A (PKA) [30] were significantly increased in WAT of these mice (figure 2*f–h*).

### 3.2. PIKO mice have higher energy expenditure

Because energy homeostasis is maintained by a balance between food intake and energy expenditure [1], we investigated the possible reasons for the decreased fat mass in PIKO mice from these two aspects. Food intake was not altered, but energy expenditure was increased as, measured by higher oxygen consumption, and the respiratory exchange ratio (RER; 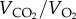) was decreased in PIKO mice (figure 2*i–k*). Although physical activity was not affected, body temperature was much higher in PIKO mice (figure 2*l,m*). Furthermore, the adipocyte volume in BAT was decreased (figure 2*n,o*), *Ucp1* and *Pgc1α* expression and UCP1 protein were increased in BAT of PIKO mice (figure 2*p,q*), and the serum NE level was increased in PIKO mice (figure 2*r*).

### 3.3. PIKO mice are resistant to high-fat diet-induced obesity

**Figure 3.**
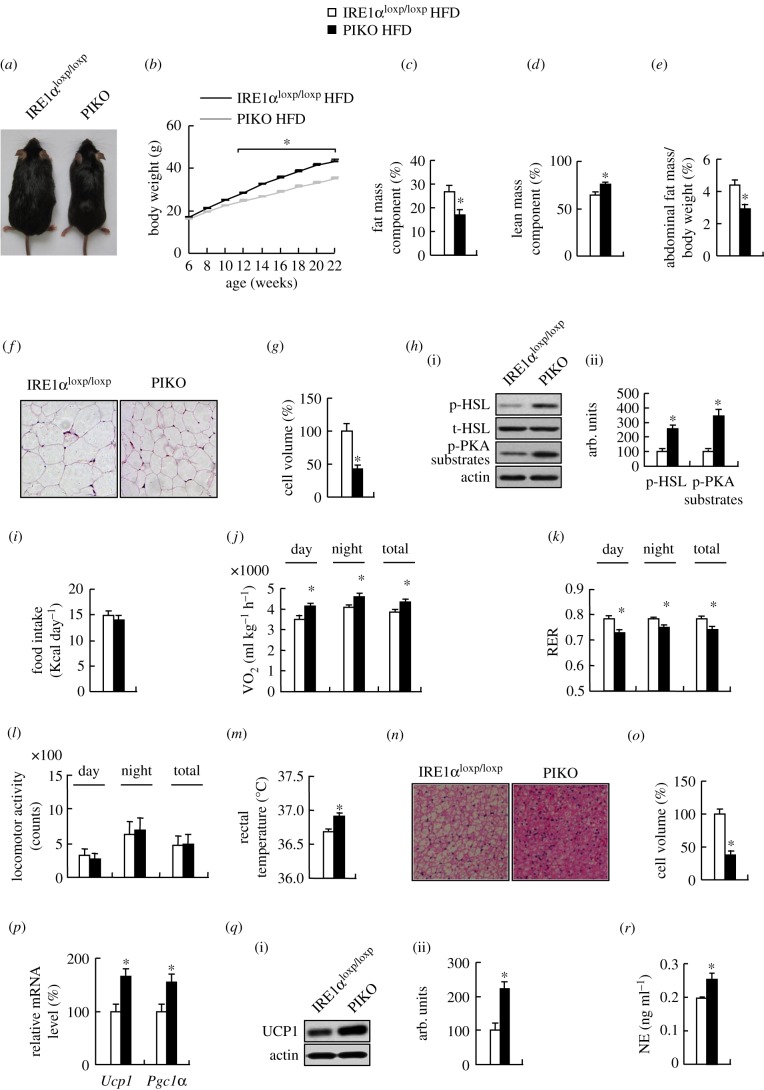
PIKO mice are resistant to HFD-induced obesity. (*a*) The gross morphology; (*b*) body weight curve; (*c*) body fat mass component; (*d*) lean mass component; (*e*) abdominal fat mass; (*f*) representative images of H&E (haematoxylin and eosin) staining of abdominal WAT sections (×10); (*g*) the relative cell volume of white adipose tissue; (*h*) p-HSL, HSL and p-PKA substrate proteins in abdominal WAT (i, western blot; ii, quantitative measurements of p-HSL and p-PKA substrates relative to the total protein or actin); (*i*) food intake; (*j*) oxygen consumption; (*k*) respiratory exchange ratio (RER, 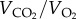); (*l*) locomotor activity; (*m*) average basal rectal temperature; (*n*) representative images of H&E staining of BAT sections (×10); (*o*) the relative cell volume of brown adipose tissue; (*p*) *Ucp1* and *Pgc1α* mRNAs in BAT; (*q*) UCP1 proteins in BAT (left, western blot; right, quantitative measurement of UCP1 relative to actin); (*r*) serum NE levels. All studies were conducted in male control mice and PIKO mice fed on a HFD for 16 weeks, starting at six weeks old. Values are means ± s.e.m. (*n* = 8–10 per group), *p < 0.05.

We then analysed the metabolic parameters in PIKO and control mice maintained on a HFD for 16 weeks. The gross morphology, body weight, fat mass component and abdominal fat mass were lower, and body lean mass component was higher in PIKO mice (figure 3*a–e*). WAT cell volume was decreased and levels of p-HSL and p-PKA substrates were increased in PIKO mice (figure 3*f–h*). In addition, PIKO mice had higher oxygen consumption and body temperature, and lower RER, with no effect on food intake and physical activity compared with control mice under HFD (figure 3*i–m*). BAT cell volume was decreased, *Ucp1* and *Pgc1α* expression and UCP1 protein were increased in BAT of PIKO mice (figure 3*n–q*), and the serum NE level was increased in PIKO mice under HFD (figure 3*r*).

### 3.4. Insulin sensitivity is improved in PIKO mice

**Figure 4.**
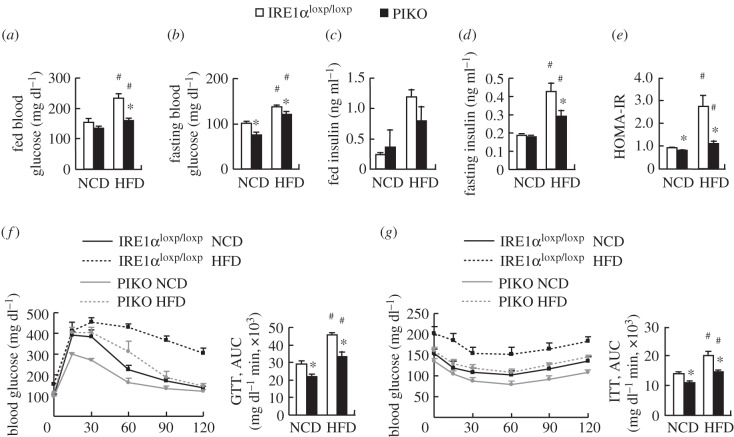
Insulin sensitivity is improved in PIKO mice. (*a–d*) Fed and fasting blood glucose and serum insulin levels; (*e*) homeostasis model assessment of insulin resistance (HOMA-IR) index; (*f–g*) GTTs and ITTs. All studies were conducted in male control and PIKO mice fed a standard chow diet (NCD) or HFD for 16 weeks, starting from six weeks old. Values are means ± s.e.m. (*n* = 8–10 per group), *p < 0.05 for the effects of PIKO mice versus control mice under the same diet; ^#^*p* < 0.05 for the effects of HFD versus standard chow diet in the same genotype mice.

Because a change in body fat component is associated with changes in insulin sensitivity [1], we investigated whether insulin sensitivity was altered in PIKO mice under standard chow diet or HFD. Although fed blood glucose and fed or fasting insulin were not changed, fasting blood glucose and HOMA-IR index were significantly decreased in PIKO mice maintained on a standard chow diet (figure 4*a–e*). Consistently, glucose tolerance and clearance were also improved significantly in PIKO mice maintained on a standard chow diet as demonstrated by GTTs and ITTs, respectively (figure 4*f,g*). The HFD-induced insulin resistance in control mice as shown by the significantly increased fed and fasting blood glucose and serum insulin and HOMA-IR index, and attenuated glucose tolerance and insulin sensitivity was, however, largely prevented in PIKO mice (figure 4*a–g*).

### 3.5. Leptin sensitivity is improved in PIKO mice

**Figure 5.**
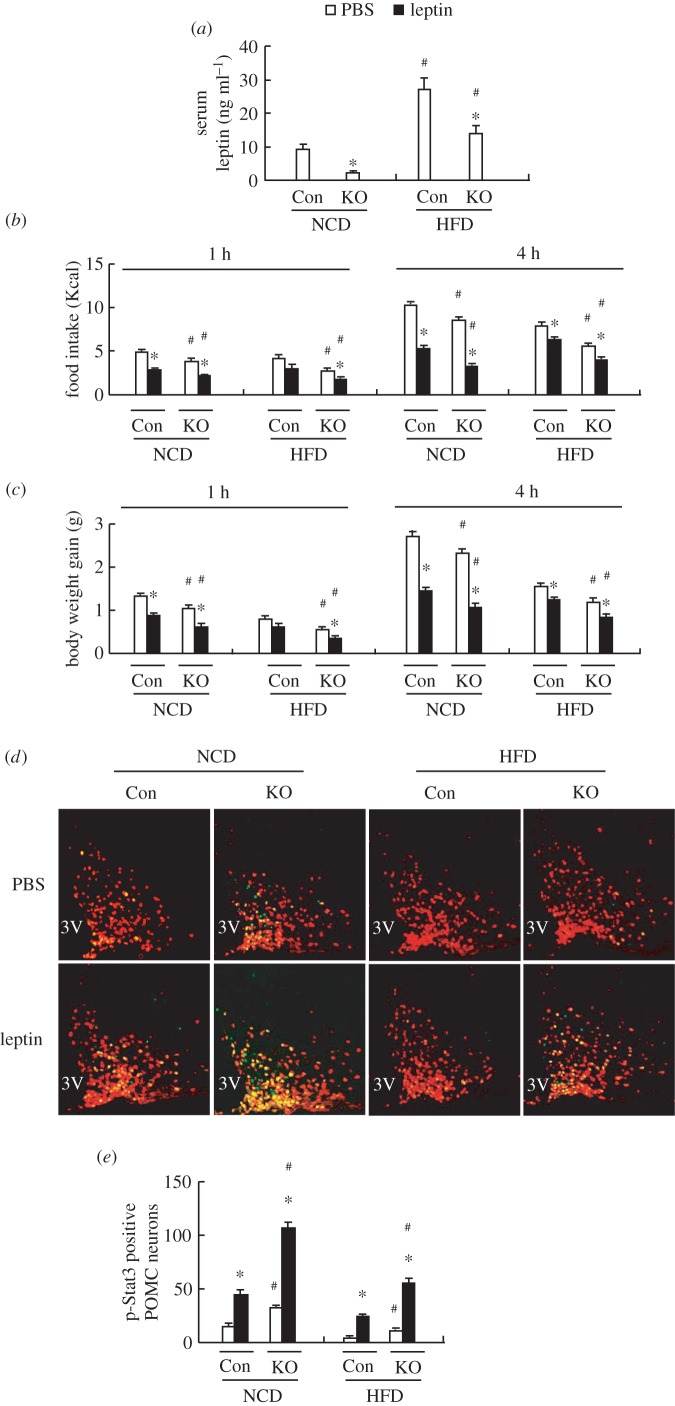
Leptin sensitivity is improved in PIKO mice. (*a*) Serum leptin levels; (*b,c*) food intake and body weight changes in mice 1 and 4 h after being injected i.p. with 3 mg kg^−1^ leptin or PBS; (*d*) representative images showing IF staining of p-STAT3 (green) in the POMC neurons (red) of mice injected i.p. with 3 mg kg^−1^ leptin or PBS for 45 min (×20); 3V, third ventricle; (*e*) statistical analysis of p-STAT3-positive POMC neurons in (*d*). All studies were conducted in male control, and PIKO mice fed a standard chow diet (NCD) or HFD for 16 weeks, starting from six weeks old. Values are means ± s.e.m. (*n* = 8–10 per group in *a*–*c*; *n* = 5 per group in *d,e*), *p < 0.05 for the effect of PIKO mice versus control mice in *a* or for the effects of leptin-treated mice versus PBS-treated mice in *b–e*, ^#^*p* < 0.05 for the effect of HFD versus standard chow diet in *a* or for the effects of PIKO mice versus control mice in *b–e*.

Reduced body fat component and increased energy expenditure imply improved leptin sensitivity in mice [31], and serum leptin levels were decreased in PIKO mice under either a standard chow diet or HFD (figure 5*a*), suggesting that leptin sensitivity was improved in PIKO mice. To test this possibility, we measured food intake and body weight 1 and 4 h following injection i.p. with 3 mg kg^−1^ leptin or PBS [22] in control and PIKO mice maintained on a standard chow diet. Leptin (1 or 4 h) injection significantly decreased food intake and body weight in control and PIKO mice; however, the extent was much more significant in PIKO mice (figure 5*b,c*). Furthermore, leptin-induced phosphorylation of signal transducer and activator of transcription 3 (p-STAT3), an indicator for leptin signalling [22], was stronger in the POMC neurons of PIKO mice compared with control mice (figure 5*d,e*). Similar effects were observed in PIKO and control mice maintained on a HFD (figure 5*b–e*).

### 3.6. Hepatic lipid accumulation is reduced in PIKO mice

**Figure 6.**
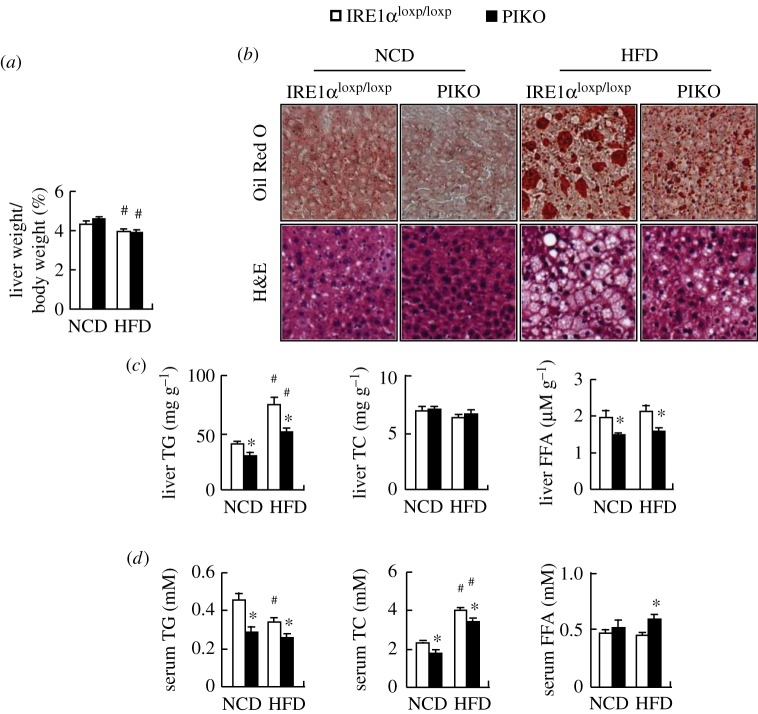
Hepatic lipid accumulation is reduced in PIKO mice. (*a*) Liver weight; (*b*) representative images of Oil Red O and H&E staining of liver sections (×10); (*c,d*) liver and serum TG, TC and FFA levels. All studies were conducted in male control, and PIKO mice fed a standard chow diet (NCD) or HFD for 16 weeks, starting from six weeks old. Values are means ± s.e.m. (*n* = 8–10 per group), *p < 0.05 for the effects of PIKO mice versus control mice under the same diet; ^#^*p* < 0.05 for the effects of HFD versus standard chow diet in the same genotype mice.

Because an alteration in fat mass is always associated with a change in lipid accumulation in liver [32], we therefore investigated whether this occurred in PIKO mice. On a standard chow diet, PIKO mice had lower hepatic lipid accumulation as demonstrated by the decreased lipid droplets shown by Oil Red O and H&E staining, reduced hepatic TG and FFAs levels, and reduced serum TG and TC levels (figure 6*b–d*). Liver weight and hepatic TC and serum FFA levels, however, were not altered in PIKO mice (figure 6*a,c,d*). HFD induced significant liver steatosis in control mice, which was largely prevented in PIKO mice (figure 6*a–d*).

### 3.7. Alpha-melanocyte-stimulating hormone production is increased in the hypothalamus of PIKO mice

**Figure 7.**
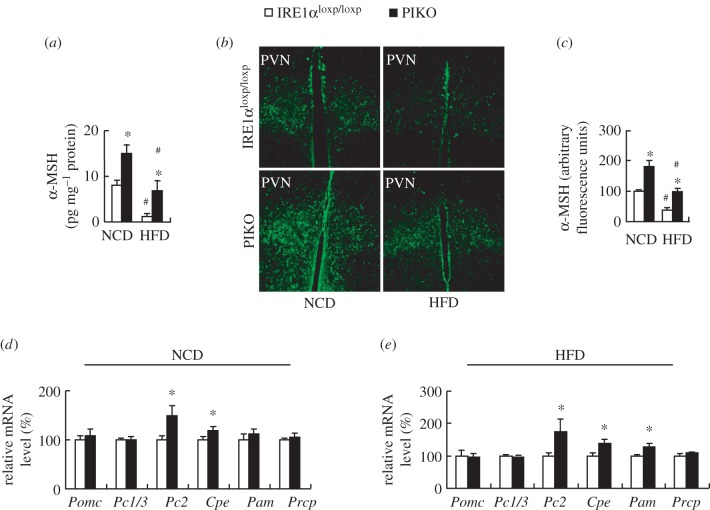
α-MSH production is increased in the hypothalamus of PIKO mice. (*a*) Relative hypothalamic α-MSH content; (*b*) representative images showing IF staining of α-MSH in the PVN (×10); (*c*) quantification analysis of α-MSH in the PVN; (*d,e*) hypothalamic *Pomc*, *Pc1/3*, *Pc2*, *Cpe*, *Pam* and *Prcp* mRNA levels. All studies were conducted in male control, and PIKO mice fed a standard chow diet (NCD) or HFD for 16 weeks, starting from six weeks old. Values are means ± s.e.m. (*n* = 8–10 per group in *a*,*d,e*; *n* = 5 per group in *b,c*), *p < 0.05 for the effects of PIKO mice versus control mice in *d* and *e* or for the effects of PIKO mice versus control mice under the same diet in *a,c*, ^#^*p* < 0.05 for the effects of HFD versus standard chow diet in the same genotype mice in *a,c*.

To explore the mechanisms underlying the lean phenotype of PIKO mice, we measured the protein levels of α-MSH, a key component vital for the regulation of obesity and energy metabolism [27], in the hypothalamus of PIKO and control mice under either a standard chow diet or HFD. Hypothalamic α-MSH levels were increased, and the staining of α-MSH in the paraventricular nucleus (PVN) of the hypothalamus was much stronger in PIKO mice compared with control mice maintained on a standard chow diet (figure 7*a–c*). HFD decreased hypothalamic α-MSH levels and weakened its staining in the PVN of control mice; however, these effects were largely blocked in PIKO mice (figure 7*a–c*). α-MSH originates from the processing of POMC by enzymes including prohormone convertase 1 (*Pc1/3*), prohormone convertase 2 (*Pc2*), carboxypeptidase E (*Cpe*), α-amidating monooxygenase (*Pam*) and prolylcarboxypeptidase (*Prcp*) [27]. Most of these genes were not changed; *Pc2* and *Cpe* expressions were increased in the hypothalamus of PIKO mice under both diets (figure 7*d,e*). In addition, the expression of feeding neuropeptides *Pomc*, Agouti-related peptide (*Agrp*), neuropeptide Y (*Npy*) and cocaine and amphetamine-related transcript (*Cart*) [27] showed no significant differences in the hypothalamus of PIKO mice and that of control mice (figure 7*d,e* and electronic supplementary material, figure S3).

## 4. Discussion

Previous works have shown that IRE1α plays an important role in ER stress [17], autophagy [15] and insulin secretion [33]. It is also indicated that IRE1α controls lipogenic gene expression and adipocyte differentiation [16,34]. The embryonic lethality in IRE1α deletion mice [35], however, results in limiting the study of loss of gene function. Recent studies by using the Cre/loxP system identified the hepatocyte-specific role of IRE1α in fatty acid β-oxidation, ketogenesis and liver regeneration [18,36]. However, the role of IRE1α in the regulation of energy homeostasis is poorly understood.

Here, we showed that knockout of IRE1α in POMC neurons decreases fat mass and protects mice from HFD-induced obesity and obesity-related metabolic disorders, the effects of which are caused by increased energy expenditure. Furthermore, the decreased fat mass is associated with increased α-MSH production in the hypothalamus of PIKO mice. We speculated that the phenotypes observed in PIKO mice were mainly caused by knockout of IRE1α in POMC neurons. POMC promoter, however, also drives cre recombinase expression in the pituitary [27,29]. There was no significant defect in the pituitary–adrenal axis observed in PIKO mice, suggesting that the pituitary–adrenal axis is unlikely to be involved in the regulation of the lean phenotype in PIKO mice.

Food intake, however, was not altered in PIKO mice compared with control mice. Consistent with our results, it is shown that pharmacological or genetic blockade of melanocortin receptors in the CNS had no effect on food intake [37,38]. As a result, the increased energy expenditure should certainly make a great contribution to the decreased fat mass in PIKO mice. We further demonstrated that the increased energy expenditure in PIKO mice was caused by induced thermogenesis in BAT, rather than a change in physical activity. BAT oxidizes fat to produce heat via increased expression of UCPs. Deletion of UCP1 induces obesity and upregulation of UCP1 increases thermogenesis and energy expenditure in mice [39]. Lipolysis in WAT is regulated by lipases, including HSL [40]. BAT and WAT have receptors for NE that are activated by hypothalamic signals via stimulation of the sympathetic nervous system (SNS) [39–41]. The expression of UCP1 in BAT and phosphorylation of HSL in WAT, as well as serum NE levels, were increased in PIKO mice, suggesting that deletion of IRE1α in POMC neurons activated the SNS and increased thermogenesis and lipolysis in PIKO mice.

It has been shown that altered body fat mass is associated with other metabolic changes, including insulin sensitivity and leptin sensitivity [42]. Insulin binds to insulin receptors expressed in different tissues to maintain normal blood glucose levels [43] and leptin binds to leptin receptors highly expressed in POMC neurons to regulate energy homeostasis and lipid metabolism [44]. Therefore, insulin and leptin are critical for the maintenance of adequate glucose and lipid metabolism [27,45,46]. Here, increased responses to insulin and leptin were observed in PIKO mice, as demonstrated by the improved glucose clearance and insulin sensitivity, and increased leptin effects and p-STAT3 staining in POMC neurons of these mice. The improved insulin sensitivity and leptin sensitivity may contribute to the lean phenotypes in PIKO mice. Lipid accumulation in liver is also associated with changes in body fat component [32]. Consistent with this, we observed that PIKO mice have reduced hepatic TG accumulation and these mice were resistant to HFD-induced liver steatosis. We hypothesized that these associated metabolic changes in PIKO mice were most likely caused by the decreased fat mass in PIKO mice. It is also, however, shown hypothalamic signals have direct effect on insulin sensitivity and lipid metabolism [24,47,48]; the possibility that IRE1α in POMC neurons might also have direct effect on these metabolic changes should not be excluded.

POMC is a prohormone that undergoes posttranslational proteolysis by several convertases to generate α-MSH [27,49]. In this study, we found that α-MSH levels were increased in the hypothalamus of PIKO mice, suggesting that the effects of IRE1α in POMC neurons on fat mass might be mediated by increased α-MSH production. Furthermore, the expression of *Pomc* and other feeding neuropeptides were not changed, whereas POMC processing enzymes including *Pc2* and *Cpe* were increased in the hypothalamus of PIKO mice, suggesting that knockout of IRE1α might increase α-MSH levels by inducing expression of POMC processing enzymes. These possibilities, however, required further investigation.

IRE1α has a kinase domain and an RNase domain in the cytosolic region, and the RNase activity induces the splicing of XBP1 [13]. Consistently, the expression of XBP1s was reduced in the ARC of PIKO mice. In contrast to the lean phenotype and the resistance to HFD-induced obesity in PIKO mice, inhibition of XBP1 in the hypothalamus results in an obese phenotype in mice [50], and activation of XBP1 in POMC neurons protects mice against HFD-induced obesity [42]. We speculated that the possible reasons for the different phenotypes observed in our study and those of the other work [42,50] could be as follows: XBP1 splicing represents one of the downstream pathways from the activation of the RNase domain of IRE1α, whereas other signalling pathways might also be activated or inhibited by the knockout of IRE1α, which may contribute to the lean phenotype in PIKO mice. For example, altered expression of hypothalamic c-Jun N-terminal kinases (JNK), as a downstream target of the kinase domain of IRE1α [17], also regulates obesity and its related diseases [51,52]. Interestingly, the expression of ER stress markers, including phosphorylated eukaryotic translation initiation factor 2α (p-eIF2α), BiP and activating transcription factor 4 (ATF4) [27] was decreased in the hypothalamus of PIKO mice under both standard chow diet and HFD (electronic supplementary material, figure S4), suggesting another possibility in explaining the effects of IRE1α deficiency in POMC neurons. The possible involvement of these signalling pathways in IRE1α regulation of energy balance requires to be studied in the future.

Taken together, we provided the first link between energy homeostasis, lipid metabolism and POMC neuronal IRE1α function. Furthermore, the effects of POMC IRE1α knockout were mediated via increased thermogenesis, possibly caused by the increased α-MSH production in the hypothalamus. This previously unidentified role of hypothalamic IRE1α also indicates a potential novel drug target in treating obesity and obesity-related metabolic disorders.

## Supplementary Material

IRE1a supplementary materials

## Data accessibility

The datasets supporting this article have been uploaded as part of the electronic supplementary material.
