# Supplementary material for: Knockout of inositol-requiring enzyme 1α in pro-opiomelanocortin neurons decreases fat mass via increasing energy expenditure
Source: Open Biol. 2016 Aug 24;6(8):160131. doi: 10.1098/rsob.160131 (PMC5008012; doi:10.1098/rsob.160131)
Supplement: IRE1a supplementary materials [file rsob160131supp1.pdf]

Knockout of IRE1α in POMC neurons decreases fat mass via increasing energy expenditure

Yuzhong Xiao [#], Tingting Xia [#], Junjie Yu, Yalan Deng, Hao Liu, Bin Liu, Shanghai Chen, Yong Liu*, and Feifan Guo*

# Y. X. and T. X. contributed equally to this work.

Key Laboratory of Nutrition and Metabolism, Institute for Nutritional Sciences, Shanghai Institute for Biological Sciences, Chinese Academy of Sciences, University of Chinese Academy of Sciences, 320 Yueyang Road, Shanghai, China.

Contact Information

*Correspondence should be addressed to Feifan Guo; E-mail: ffguo@sibs.ac.cn,

Address: 320 Yueyang Road, Shanghai, China 200031 or Yong Liu; E-mail: liuy@sibs.ac.cn, Address: 320 Yueyang Road, Shanghai, China 200031

E-mail: ffguo@sibs.ac.cn; Tel: 0086 21 5492 0250; Fax: 0086 21 5492 0291

liuy@sibs.ac.cn; Tel: 0086 21 5492 0244; Fax: 0086 21 5492 0291

Running title: POMC IRE1α regulates obesity

Supplementary Figure 1. The distribution and counts of POMC neurons are not affected by IRE1α deletion in POMC neurons. (A) Representative immunofluorescence images of the arcuate nucleus (ARC), POMC neurons (red) (20×) 3V: third ventricle; (B) POMC neurons area; (C) POMC neurons distribution and number throughout ARC. All studies were conducted in 8-weeks old male POMC-Cre/Ai9 and PIKO/Ai9 mice maintained on a standard chow diet. Values are means ± SEM (n = 4/group).

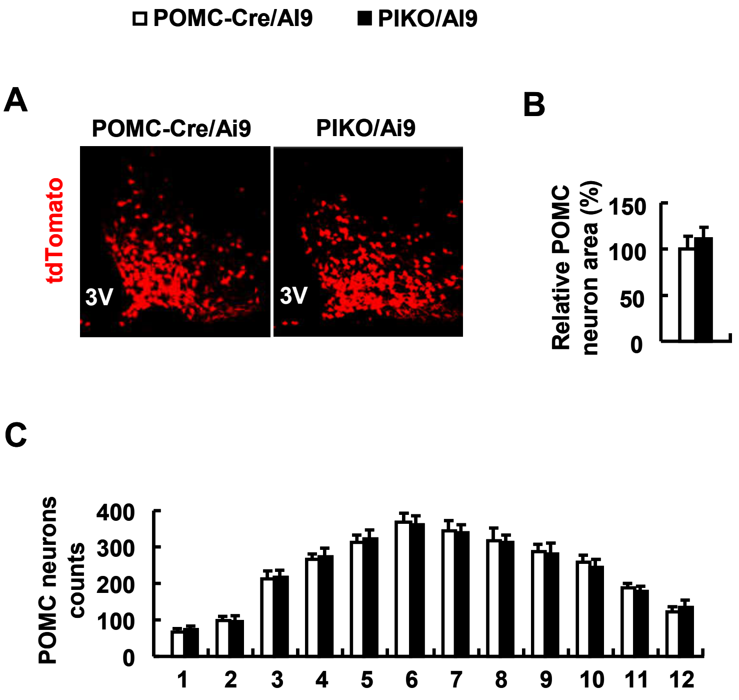

Supplementary Figure 2. The function of the pituitary-adrenal axis is not affected by IRE1α deletion in POMC neurons. (A) Serum corticosterone levels under basal and stressed conditions; (B) Serum growth hormone levels; (C) Expression of representative pituitary genes in the pituitary; (D) Representative images of the pituitary, POMC expressing cells (red) (4×); (E) pituitary area. Studies

were conducted in 8-weeks old male control and PIKO mice maintained on a standard chow diet in A-C or in 8-weeks old male POMC-Cre/Ai9 mice and PIKO/Ai9 mice maintained on a standard chow diet in D and E. Values are means ± SEM (n =6-7/group in A-C and n= 4/group in D-E).

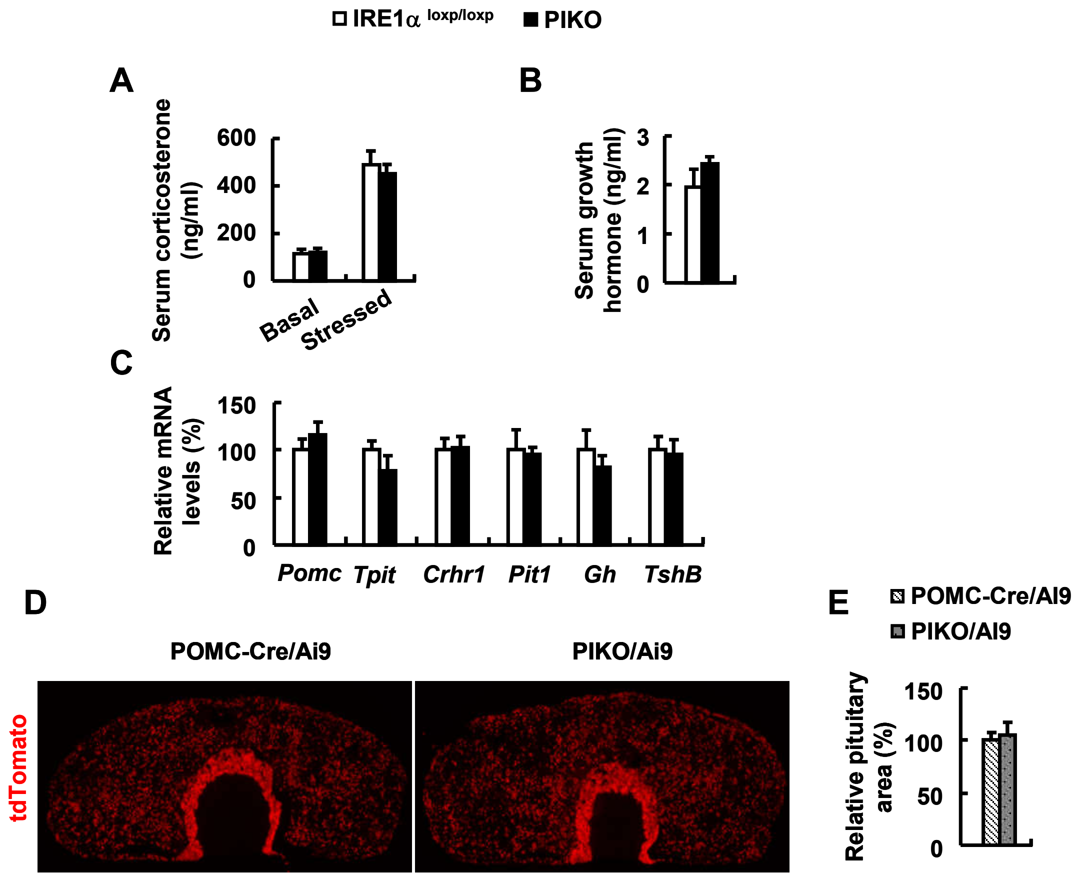

Supplementary figure 3. The expression of feeding neuropeptides *Agrp, Npy* and *Cart* are not altered in the hypothalamus of PIKO mice. (A and B) hypothalamic *Agrp, Npy* and *Cart* mRNA levels. All studies were conducted in male control and PIKO mice fed on a standard chow diet (NCD) or high-fat diet (HFD) for 16 weeks, starting from 6-weeks old. Values are means ± SEM (n = 8-10/group).

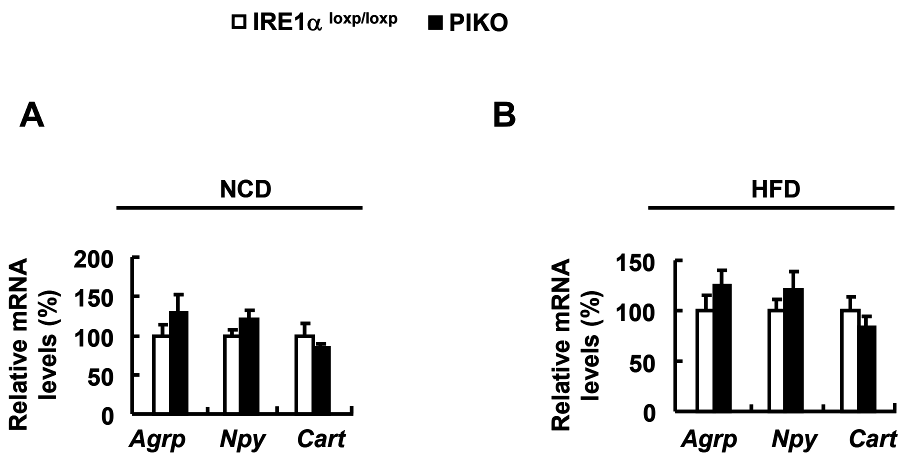

Supplementary figure 4. The ER stress is ameliorated in the hypothalamus of PIKO mice under both a standard chow diet and high-fat diet (HFD). (A) p-eIF2α, BiP and ATF4 proteins in the hypothalamus (*left*, western blot; *right*, quantitative measurement of p-eIF2α, BiP and ATF4 relative to total protein or actin); (B) Representative images showing immunofluorescence staining of ATF4 (green) in the arcuate nucleus (ARC) of mice (20×), 3V: third ventricle; (C) Quantification analysis of ATF4 in ARC. All studies were conducted in male control and PIKO mice fed on a standard chow diet (NCD) or HFD for 16 weeks, starting from 6-weeks old. Values are means ± SEM (n = 8-10/group in A, n = 4/group in B-C), *P < 0.05 for the effects of PIKO mice versus control mice in A and C, $^{\#}$P < 0.05 for the effects of HFD versus standard chow diet in the same genotype mice in A and C.

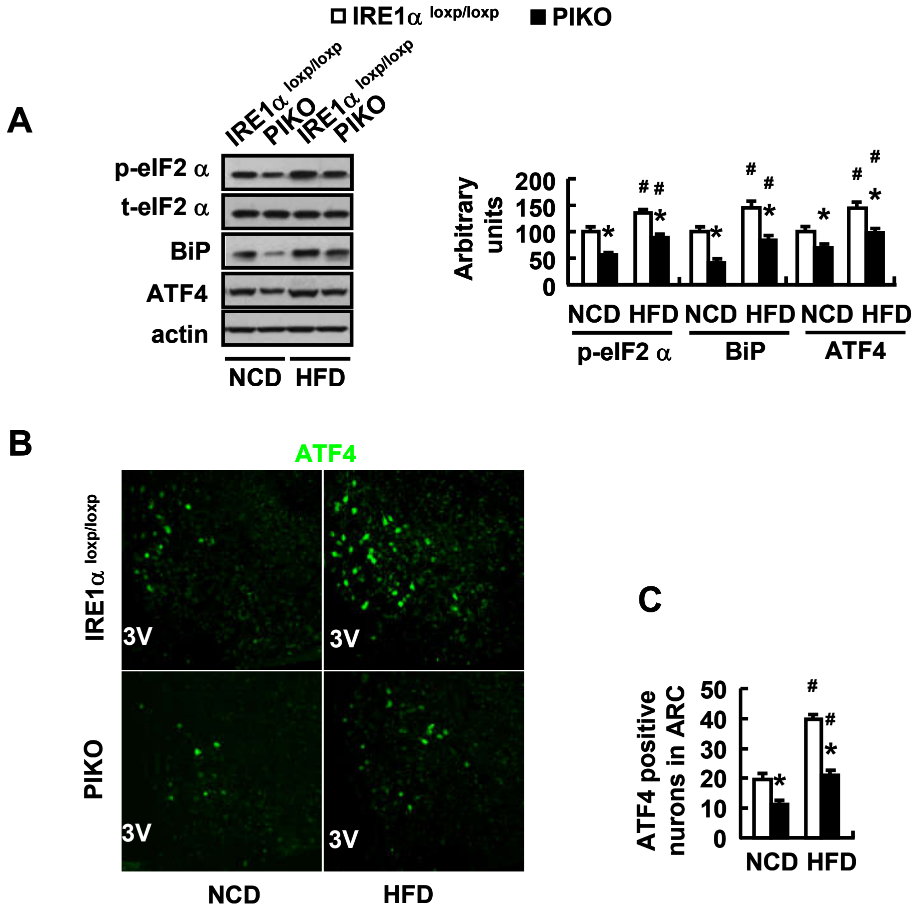

IRE1α loxp/loxp
PIKO
A
p-eIF2 α
t-eIF2 α
BiP
ATF4
actin
NCD
HFD
Arbitrary units
200
150
100
50
0
NCD HFD NCD HFD NCD HFD
p-eIF2 α
BiP
ATF4
B
ATF4
3V
NCD
HFD
C
ATF4 positive nurons in ARC
50
40
30
20
10
0
NCD HFD
